# Kidney Stone History and Survival Outcomes in Upper Tract Urothelial Carcinoma

**DOI:** 10.1001/jamanetworkopen.2025.41054

**Published:** 2025-11-03

**Authors:** Bor-En Jong, Hsi-Chin Wu, Wen-Chi Chen, Wen-Jeng Wu, Ching-Chia Li, Yi-Hsin Lu, Chen-Han Wilfred Wu, Yao-Chou Tsai

**Affiliations:** 1 Division of Urology, Department of Surgery, Taipei Tzu Chi Hospital, The Buddhist Medical Foundation, New Taipei City, Taiwan; 2 School of Medicine, Buddhist Tzu Chi University, Hualien, Taiwan; 3 Department of Genetics and Genome Sciences, Case Western Reserve University School of Medicine and University Hospitals, Cleveland, Ohio; 4 Department of Urology, Case Western Reserve University School of Medicine and University Hospitals, Cleveland, Ohio; 5 Department of Urology, China Medical University Hospital, Taichung, Taiwan; 6 School of Medicine, China Medical University, Taichung, Taiwan; 7 Department of Urology, China Medical University Beigang Hospital, Yunlin, Taiwan; 8 Graduate Institute of Integrated Medicine, College of Chinese Medicine, China Medical University, Taichung, Taiwan; 9 Department of Urology, Kaohsiung Medical University Hospital, Kaohsiung, Taiwan; 10 Department of Urology, School of Medicine, College of Medicine, Kaohsiung Medical University, Kaohsiung, Taiwan; 11 Graduate Institute of Clinical Medicine, College of Medicine, Kaohsiung Medical University, Kaohsiung, Taiwan; 12 Department of Urology, Kaohsiung Medical University Gangshan Hospital, Kaohsiung, Taiwan

## Abstract

**Importance:**

Upper tract urothelial carcinoma (UTUC) is aggressive, and whether a history of urinary tract stones influences survival after radical nephroureterectomy (RNU) is unclear.

**Objective:**

To determine the association between urinary stone history and oncological outcomes in patients with UTUC undergoing RNU.

**Design, Setting, and Participants:**

This multicenter retrospective cohort study was part of the Taiwan UTUC Registry Study, a multicenter registry encompassing 21 tertiary and regional hospitals throughout Taiwan. Medical records from September 1, 1988, to December 31, 2023, were reviewed; follow-up continued through December 31, 2024, and data were analyzed from January 15 to March 30, 2025. Participants were adults with histologically confirmed UTUC who underwent RNU.

**Exposure:**

Documented history of urinary tract stones identified via medical records.

**Main Outcomes and Measures:**

Primary outcomes were cancer-specific survival (CSS) and disease-free survival (DFS). Secondary outcomes were overall survival (OS) and bladder recurrence–free survival (BRFS). Hazard ratios (HRs) with 95% CIs were estimated using Cox proportional hazards models adjusted with overlap weighting.

**Results:**

Of 5824 registry patients, 3414 patients (mean [SD] age, 68.2 [10.5] years; 1957 female [57.3%]) met inclusion criteria. Among these, 169 (4.9%) had a history of urinary stones. Median (IQR) follow-up was 53.86 (23.72-92.50) months. Patients with history of urinary stones, compared with those without, had higher rates of metastasis (25 patients [14.8%] vs 233 patients [7.2%]) and UTUC-specific death (47 patients [27.8%] vs 599 patients [18.5%]). After overlap weighting, stone history was independently associated with worse CSS (HR, 1.83; 95% CI, 1.35-2.47; *P* < .001) and DFS (HR, 1.69; 95% CI, 1.29-2.21; *P* < .001). Stone history was not significantly associated with OS (HR, 1.18; 95% CI, 0.94-1.48) or BRFS (HR, 1.09; 95% CI, 0.86-1.37).

**Conclusions and Relevance:**

In this cohort study of patients with UTUC who underwent radical nephroureterectomy, a history of urinary tract stones was associated with inferior CSS and DFS, suggesting that patients with UTUC and stone history may represent a higher-risk subgroup that could warrant intensified surveillance and consideration of tailored adjuvant therapy.

## Key Points

**Question:**

Is a history of urinary tract stones associated with survival outcomes among patients with upper tract urothelial carcinoma (UTUC)?

**Findings:**

In this cohort study of 3414 patients with UTUC, a history of urinary stones was associated with significantly worse cancer-specific survival and disease-free survival, even after adjusting for confounders using overlap weighting.

**Meaning:**

These findings suggest that patients with UTUC and stone history may represent a high-risk subgroup requiring intensified surveillance and potentially tailored adjuvant therapy.

## Teaser

This cohort study examines the association of history of urinary tract stones with survival outcomes among patients with upper tract urothelial carcinoma after radical nephroureterectomy.

## Introduction

Urinary tract stone disease is one of the most prevalent urological conditions, affecting millions of people worldwide.^1^ This condition is characterized by the formation of crystalline deposits in the renal system and can cause considerable morbidity, including recurrent pain, urinary obstruction, infections, and, in severe cases, kidney dysfunction. Beyond these immediate effects, recent studies suggest a link between nephrolithiasis and upper tract urothelial carcinoma (UTUC).^2,3,4,5^ This possible association raises new concerns about the role of stone disease in carcinogenesis and its influence on long-term oncological outcomes.

UTUC is a relatively rare yet aggressive malignant neoplasm and accounts for only 5% to 10% of urothelial cancers.^6^ In contrast to bladder cancer, UTUC is often diagnosed at more advanced stages and carries a poor prognosis with fewer therapeutic options.^7^ Established prognostic factors, including tumor stage, histological grade, lymphovascular invasion, and nodal involvement, are critical for estimating disease progression and survival.^8,9^ However, additional factors that affect UTUC onset and prognosis have yet to be fully defined. Because urinary stone disease has been suggested as a potential risk factor, clarifying their relationship to UTUC prognosis is clinically relevant.

Epidemiological evidence supports an association between stones and incident UTUC. A meta-analysis reported more than a 2-fold increased UTUC risk among individuals with prior stones (pooled risk ratio, 2.14; 95% CI, 1.35-3.40),^10^ and large population-based studies have shown similar patterns.^2,3,4^ Biological explanations include chronic mechanical irritation of the urothelium, persistent inflammation, oxidative stress, and DNA damage.^5^ Urinary obstruction caused by stones might also prolong urothelial exposure to carcinogens, further promoting oncogenic changes.^11^

Although previous research has largely examined the increased incidence of UTUC in patients with urinary stones,^2,3,4^ the influence of stone disease on oncological outcomes, like recurrence, metastasis, and survival, has not been thoroughly investigated. Given UTUC’s aggressive clinical course, identifying additional prognostic factors that drive disease progression is essential. Existing prognostic models primarily focus on tumor-specific characteristics,^12^ and risk factors, such as urinary stones, have not been formally integrated into survival analyses.

In this study, we used the Taiwan UTUC Registry Database, a large multi-institutional registry, to evaluate the prognostic association of urinary stone history in patients with UTUC undergoing radical nephroureterectomy (RNU). To our knowledge, this is the first comprehensive investigation into the association of urinary stone disease with UTUC outcomes. By defining the magnitude and direction of these associations, this study aims to refine risk stratification and inform clinical decision-making and management strategies for patients with UTUC who have a history of urinary stones.

## Methods

### Study Design and Setting

This retrospective cohort study was approved by the institutional review board of Taipei Tzu Chi Hospital, Buddhist Tzu Chi Medical Foundation, with a waiver of informed consent because the study used deidentified data and posed minimal risk. This study is reported in adherence with the Strengthening the Reporting of Observational Studies in Epidemiology (STROBE) reporting guideline. Data came from the Taiwan UTUC Registry Database, which aggregates prospectively maintained records from 21 tertiary or regional hospitals across Taiwan.^13^ The registry includes patients treated from September 1, 1988, to December 31, 2023, and survival outcomes, including vital status and recurrence, were updated through December 31, 2024.

### Participants and Follow-Up

All adults with histologically confirmed UTUC who underwent RNU in participating health centers were screened. Patients were excluded if they received nonradical treatments (eg, endoscopic or segmental resection), had incomplete data for key covariates required for propensity score or survival analyses, or lacked outcome follow-up. Reasons for exclusion due to missingness and loss to follow-up are detailed in eFigure 1 in Supplement 1. Follow-up began at the date of RNU and continued until the event of interest or last contact; patients alive and event-free were censored at database close.

### Exposure, Outcomes, Covariates, and Data Sources

The exposure was a history of urinary tract stones, abstracted from medical records as a binary variable (yes or no). Outcomes were overall survival (OS), cancer-specific survival (CSS), disease-free survival (DFS; time to first recurrence, metastasis, or UTUC death), and bladder recurrence–free survival (BRFS; time to first intravesical recurrence). Baseline covariates included demographics (age at surgery, sex), clinical factors (smoking, herbal supplement use, end-stage kidney disease [ESKD] or chronic kidney insufficiency [CKI], and any previous urothelial malignant neoplasms). Tumor-related characteristics included histological subtype (urothelial vs variant), anatomical location (renal pelvis, ureter, bladder cuff, or multiple sites), tumor size (<2 cm vs ≥2 cm), pathological stage (stage 0a/0is/I, stage II, stage III-IV), and tumor grade (low vs high). Additional pathological parameters, such as lymphovascular invasion, tumor necrosis, and surgical margin status, were obtained from pathology reports. Surgical approach, surgical access, and perioperative intravesical therapy were also recorded. Trained investigators abstracted standardized variables from electronic and paper medical records; pathology features were recorded from institutional reports; outcomes were ascertained from clinic notes, hospital records, and death documentation, using uniform registry definitions across centers.

### Statistical Analysis

Continuous variables were reported as means and SDs or medians with IQRs, depending on data distribution, and compared using the *t* test or Wilcoxon rank-sum test as appropriate. Categorical variables were presented as frequencies and percentages, with Fisher exact test used for comparisons. To address confounding, we used 2 complementary approaches. First, a directed acyclic graph specified a minimal adjustment set of baseline confounders for conventional Cox models (age, sex, smoking, herbal supplement use, and ESKD or CKI) (eFigure 2 in Supplement 1). Second, because only approximately 5% of the cohort had a stone history and the exposure groups were markedly imbalanced, we applied propensity score overlap weighting to improve empirical comparability. Full details of the propensity score model and overlap weighting are provided in the eMethods in Supplement 1.^14,15^ This approach down-weights individuals at the extremes of the propensity score distribution, thus emphasizing patients at clinical equipoise and mimicking the population of a pragmatic randomized trial.^16^ Fine-Gray subdistribution models and plotted cumulative incidence functions to account for competing risks. Because propensity methods require fully observed covariates, a complete-case approach was used; exclusions for missingness and lack of follow-up are detailed in eFigure 1 in Supplement 1. The study size reflected all eligible registry patients after prespecified exclusions. *P* values were 2-sided, and statistical significance was set at *P* ≤ .05. All analyses were performed using R software (R Foundation for Statistical Computing) from January 15 to March 30, 2025. 

## Results

### Participants

Of 5824 registry patients, 1461 were excluded because they did not undergo RNU and 949 were excluded for incomplete covariate data. A total of 3414 patients (mean [SD] age, 68.2 [10.5] years; 1957 female [57.3%]) underwent RNU for UTUC and were included in analysis. Of these, 169 (4.9%) had a history of urinary tract stones. Median (IQR) follow-up was 53.9 (23.7-92.5) months.

### Baseline Characteristics

Table 1 summarizes key baseline characteristics stratified by history of urinary stones. Compared with 3245 patients without stones, those with a history of urinary stones were more frequently male (94 patients [55.6%] vs 1362 patients [42.0%]), had higher rates of multifocal tumors (61 patients [36.1%] vs 831 patients [25.6%]), were more like to have tumor size 2 cm or greater (124 patients [73.4%] vs 2165 patients [66.7%]), and were more likely to have preoperative hydronephrosis (138 patients [81.7%] vs 1817 patients [56.0%]). Current or former smoking was also more prevalent among patients with a history of stones (43 patients [25.4%] vs 534 patients [16.5%]). Full baseline characteristics are reported in eTable 1 in Supplement 1.

**Table 1.** Patient Characteristic Baseline

Variables	Patients, No. (%) (N = 3414)		*P* value	SMD
	No stone history (n = 3245)	Stone history (n = 169)		
Sex				
Male	1363 (42.0)	94 (55.6)	.001	0.275
Female	1882 (58.0)	75 (44.4)		
Age, mean (SD), y	68.21 (10.52)	67.83 (9.34)	.64	0.039
Smoking				
No	2711 (83.5)	126 (74.6)	.003	0.222
Yes	534 (16.5)	43 (25.4)		
Herbal supplements				
No	3048 (93.9)	162 (95.9)	.39	0.088
Yes	197 (6.1)	7 (4.1)		
ESKD or CKI				
No	1168 (36.0)	69 (40.8)	.23	0.100
Yes	2077 (64.0)	100 (59.2)		
Bladder cancer history				
No	2984 (92.0)	163 (96.4)	.048	0.193
Yes	261 (8.0)	6 (3.6)		
Cell type				
Urothelial	2917 (89.9)	153 (90.5)	.43	0.141
UC with variants	296 (9.1)	16 (9.5)		
Others	32 (1.0)	0		
Location				
Renal pelvis	1374 (42.3)	64 (37.9)	.02	0.244
Ureter	1029 (31.7)	44 (26.0)		
Bladder cuff	11 (0.3)	0		
Multiple locations	831 (25.6)	61 (36.1)		
Size, cm	831 (25.6)	44 (26.0)		
<2			.002	0.363
≥2	2165 (66.7)	124 (73.4)		
Missing	249 (7.7)	1 (0.6)		
Pathological stage				
0a/0is/I	1347 (41.5)	72 (42.6)	.91	0.034
II	617 (19.0)	30 (17.8)		
III-IV	1281 (39.5)	67 (39.6)		
Grade				
Low	375 (11.6)	24 (14.2)	.08	0.209
High	2693 (83.0)	142 (84.0)		
Missing	177 (5.5)	3 (1.8)		
CIS				
No	2505 (77.2)	137 (81.1)	.28	0.095
Yes	740 (22.8)	32 (18.9)		
LVI				
No	2625 (80.9)	130 (76.9)	.24	0.097
Yes	620 (19.1)	39 (23.1)		
Surgical margin				
Free	3109 (95.8)	161 (95.3)	.88	0.026
Positive	136 (4.2)	8 (4.7)		
Preoperation urine cytology				
Negative	759 (23.4)	26 (15.4)	.09	0.214
Atypia	544 (16.8)	34 (20.1)		
Positive	809 (24.9)	42 (24.9)		
No cytology	1133 (34.9)	67 (39.6)		
Preoperative hydronephrosis				
No	1428 (44.0)	31 (18.3)	<.001	0.577
Yes	1817 (56.0)	138 (81.7)		
Tumor necrosis				
No	2767 (85.3)	130 (76.9)	.004	0.214
Yes	478 (14.7)	39 (23.1)		
Chemotherapy type				
No	2307 (71.1)	116 (68.6)	.53	0.115
Neoadjuvant	86 (2.7)	3 (1.8)		
Adjuvant	598 (18.4)	32 (18.9)		
Salvage or palliative	254 (7.8)	18 (10.7)		

Abbreviations: CIS, urothelial carcinoma in situ; CKI, chronic kidney insufficiency; ESKD, end-stage kidney disease; LVI, lymphovascular invasion; SMD, standardized mean difference; UC, urothelial carcinoma.

### Outcome Data and Unadjusted Results

In unadjusted comparisons, stone history was associated with less favorable outcomes compared with patients with no stone history (eTable 1 in Supplement 1). Metastasis occurred in 25 patients (14.8%) vs 233 patients (7.2%) (*P* < .001), as did UTUC-specific death (47 patients [27.8%] vs 599 patients [18.5%]; *P* = .003), and the proportion remaining disease-free was lower among patients with history of stones (110 patients [65.1%] vs 2409 patients [74.2%]; *P* = .01). Overall mortality and bladder recurrence did not differ meaningfully between groups. Among patients who died of UTUC, the median (IQR) time from diagnosis to cancer-specific death was 19.42 (IQR, 9.07-38.27) months.

### Adjusted Results

Directed acyclic graph–based Cox models adjusting for age, sex, smoking, herbal supplement use, and ESKD or CKI found that stone history was associated with inferior CSS (HR, 1.56; 95% CI, 1.16-2.10) and shorter DFS (HR, 1.39; 95% CI, 1.06-1.80) (Table 2). Associations with OS (HR, 1.11; 95% CI, 0.88-1.39) and BRFS (HR, 1.01; 95% CI, 0.75-1.36) were not observed. Female sex was associated with lower hazards for OS (HR, 0.77; 95% CI, 0.69-0.86) and BRFS (HR, 0.63; 95% CI, 0.55-0.73). Older age was associated with increased risk across all end points. ESKD and CKI were associated with to higher all-cause mortality (OS: HR, 1.43; 95% CI, 1.28-1.60) without clear associations for CSS, DFS, or BRFS.

**Table 2.** Multivariate Survival Analysis Based on DAG

Variable	OS		CSS		DFS		BRFS	
	HR (95% CI)	*P* value	HR (95% CI)	*P* value	HR (95% CI)	*P* value	HR (95% CI)	*P* value
History of stone	1.11 (0.88-1.39)	.40	1.56 (1.16-2.10)	.003	1.39 (1.06-1.80)	.02	1.01 (0.75-1.36)	.94
Gender								
Male	1 [Reference]	NA	1 [Reference]	NA	1 [Reference]	NA	1 [Reference]	NA
Female	0.77 (0.69-0.86)	<.001	0.85 (0.71-1.01)	.06	0.89 (0.76-1.03)	.12	0.63 (0.55-0.73)	<.001
Age, per 1-y increase	1.04 (1.03-1.04)	<.001	1.02 (1.01-1.03)	<.001	1.02 (1.01-1.03)	<.001	1.02 (1.01-1.03)	<.001
Smoking	0.96 (0.83-1.11)	.57	1.13 (0.91-1.40)	.28	1.16 (0.97-1.40)	.11	0.99 (0.83-1.18)	.91
Herbal supplement use	0.87 (0.70-1.08)	.22	1.09 (0.80-1.48)	.59	0.92 (0.70-1.21)	.56	0.74 (0.56-0.99)	.04
ESKD or CKI	1.43 (1.28-1.60)	<.001	1.15 (0.97-1.35)	.10	1.07 (0.94-1.23)	.31	1.03 (0.90-1.17)	.69

Abbreviations: BRFS, bladder recurrence–free survival; CKI, chronic kidney insufficiency; CSS, cancer-specific survival; DFS, disease-free survival; ESKD, end-stage kidney disease; HR, hazard ratio; NA, not applicable; OS, overall survival.

In overlap-weighted multivariable Cox models, history of urinary stones was associated with inferior CSS (HR, 1.83, 95% CI, 1.35-2.47; *P* < .001) and shorter DFS (HR, 1.69; 95% CI, 1.29-2.21; *P* < .001) (Table 3). The association with OS was borderline (HR, 1.26; 95% CI, 1.00-1.60; *P* = .05), and no association was observed for BRFS (HR, 1.11; 95% CI, 0.82-1.49; *P* = .49).

**Table 3.** Multivariate Survival Analysis With Overlap Weighted

Variable	OS		CSS		DFS		BRFS	
	HR (95% CI)	*P* value	HR (95% CI)	*P* value	HR (95% CI)	*P* value	HR (95% CI)	*P* value
History of stone	1.26 (1.00-1.60)	.05	1.83 (1.35-2.47)	<.001	1.69 (1.29-2.21)	<.001	1.11 (0.82-1.49)	.49
Female sex	0.82 (0.74-0.91)	<.001	NA	NA	NA	NA	0.67 (0.59-0.77)	<.001
Age, per 1-y increase	1.04 (1.03-1.04)	<.001	1.02 (1.01-1.03)	<.001	1.02 (1.02-1.03)	<.001	1.02 (1.01-1.03)	<.001
Cell type								
Urothelial	1 [Reference]	NA	1 [Reference]	NA	1 [Reference]	NA	1 [Reference]	NA
UC with variants	1.14 (0.95-1.36)	.16	1.20 (0.94-1.53)	.14	1.13 (0.92-1.40)	.25	NA	NA
Others	1.62 (1.06-2.48)	.03	1.74 (1.03-2.97)	.04	1.88 (1.20-2.95)	.006	NA	NA
Side								
Left	NA	NA	NA	NA	1 [Reference]	NA	1 [Reference]	NA
Right	NA	NA	NA	NA	0.83 (0.72-0.95)	.006	0.86 (0.76-0.98)	.03
Both	NA	NA	NA	NA	1.34 (0.71-2.52)	.37	0.79 (0.42-1.49)	.47
Location								
Renal pelvis	1 [Reference]	NA	1 [Reference]	NA	1 [Reference]	NA	1 [Reference]	NA
Ureter	1.05 (0.92-1.19)	.49	1.11 (0.90-1.36)	.33	1.12 (0.94-1.32)	.21	1.26 (1.07-1.48)	.005
Bladder cuff	1.16 (0.55-2.46)	.70	1.88 (0.70-5.09)	.21	1.07 (0.44-2.62)	.89	1.04 (0.43-2.56)	.92
Multiple	1.34 (1.18-1.52)	<.001	1.46 (1.21-1.76)	<.001	1.39 (1.18-1.64)	<.001	1.69 (1.44-1.98)	<.001
Size, cm								
<2	1 [Reference]	NA	1 [Reference]	NA	1 [Reference]	NA	1 [Reference]	NA
≥2	1.12 (0.97-1.29)	.13	1.91 (1.47-2.48)	<.001	1.56 (1.27-1.92)	<.001	1.09 (0.93-1.28)	.29
Not available	2.46 (1.97-3.08)	<.001	3.78 (2.61-5.48)	<.001	4.09 (2.99-5.60)	<.001	2.42 (1.78-3.29)	<.001
Pathological stage								
Stage 0a/0is/I	1 [Reference]	NA	1 [Reference]	NA	1 [Reference]	NA	1 [Reference]	NA
Stage II	1.20 (1.02-1.40)	.03	1.90 (1.42-2.54)	<.001	1.88 (1.48-2.39)	<.001	1.17 (0.99-1.40)	.07
Stage III-IV	1.94 (1.70-2.22)	<.001	4.33 (3.38-5.53)	<.001	4.18 (3.41-5.12)	<.001	1.31 (1.11-1.54)	.001
Grade								
Low	1 [Reference]	NA	1 [Reference]	NA	1 [Reference]	NA	1 [Reference]	NA
High	1.15 (0.95-1.39)	.14	1.85 (1.21-2.85)	.005	1.73 (1.24-2.42)	.001	NA	NA
Not available	1.33 (1.02-1.74)	.03	1.73 (0.99-3.03)	.06	1.21 (0.75-1.93)	.44	NA	NA
Smoker	NA	NA	NA	NA	1.21 (1.03-1.44)	.02	NA	NA
ESKD or CKI	1.33 (1.18-1.49)	<.001	NA	NA	NA	NA	NA	NA
Previous nephroureterectomy UC	1.30 (0.96-1.75)	.09	NA	NA	NA	NA	NA	NA
Previous kidney-sparing surgery UTUC	NA	NA	NA	NA	3.15 (1.56-6.38)	.001	2.88 (1.52-5.48)	.001
Bladder cancer	NA	NA	NA	NA	NA	NA	1.50 (1.21-1.87)	<.001
CIS	NA	NA	NA	NA	0.82 (0.70-0.97)	.02	NA	NA
LVI	1.57 (1.38-1.79)	<.001	1.79 (1.50-2.13)	<.001	1.75 (1.50-2.03)	<.001	1.36 (1.13-1.63)	.001
Surgical margin								
Free	1 [Reference]	NA	1 [Reference]	NA	1 [Reference]	NA	NA	NA
Positive	2.25 (1.82-2.77)	<.001	2.61 (2.02-3.37)	<.001	2.41 (1.92-3.02)	<.001	NA	NA
Preoperation urine cytology								
Negative	1 [Reference]	NA	NA	NA	NA	NA	NA	NA
Atypia	0.82 (0.68-0.98)	.03	NA	NA	NA	NA	NA	NA
Positive	1.04 (0.89-1.21)	.66	NA	NA	NA	NA	NA	NA
No cytology	1.02 (0.88-1.19)	.81	NA	NA	NA	NA	NA	NA
Tumor necrosis	1.20 (1.04-1.38)	.01	NA	NA	NA	NA	NA	NA
RNU method								
Open	NA	NA	NA	NA	NA	NA	1 [Reference]	NA
Laparoscopic hand-assisted	NA	NA	NA	NA	NA	NA	1.13 (0.94-1.36)	.19
Robot assisted	NA	NA	NA	NA	NA	NA	1.98 (1.52-2.57)	<.001
Laparoscopy	NA	NA	NA	NA	NA	NA	1.55 (1.29-1.87)	<.001
RNU access								
Transperitoneal	NA	NA	NA	NA	NA	NA	1 [Reference]	NA
Retroperitoneal	NA	NA	NA	NA	NA	NA	1.23 (1.07-1.42)	.003
Postoperation intravesical CT instillation	NA	NA	NA	NA	NA	NA	1.34 (1.03-1.73)	.03

Abbreviations: BRFS, bladder recurrence–free survival; CIS, urothelial carcinoma in situ; CKI, chronic kidney insufficiency; CSS, cancer-specific survival; CT, chemotherapy; DFS, disease-free survival; ESKD, end-stage kidney disease; HR, hazard ratio; LVI, lymphovascular invasion; NA, not applicable; OS, overall survival; RNU, radical nephroureterectomy; UC, urothelial carcinoma; UTUC, upper tract urothelial carcinoma.

### Kaplan-Meier Survival Analyses

**Figure 1.**
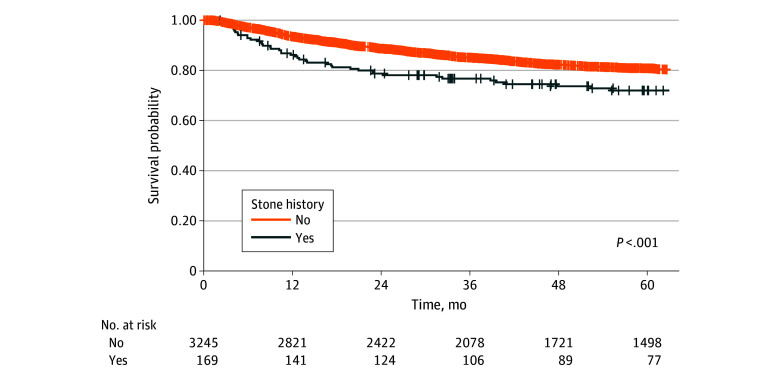
Cancer-Specific Survival in the Unweighted Cohort, Stratified by Urinary-Stone History Crosses indicate censored patients. A history of urinary stones was associated with significantly worse cancer-specific survival (*P* < .001).

Kaplan-Meier curves for CSS are depicted in Figure 1 (unweighted) and eFigure 3 in Supplement 1 (overlap weighted). In the unweighted analysis, a history of urinary stones was associated with significantly lower survival probabilities. Following overlap weighting, this survival gap remained significant, reinforcing stone disease as factor associated with adverse cancer-specific outcomes in UTUC.

**Figure 2.**
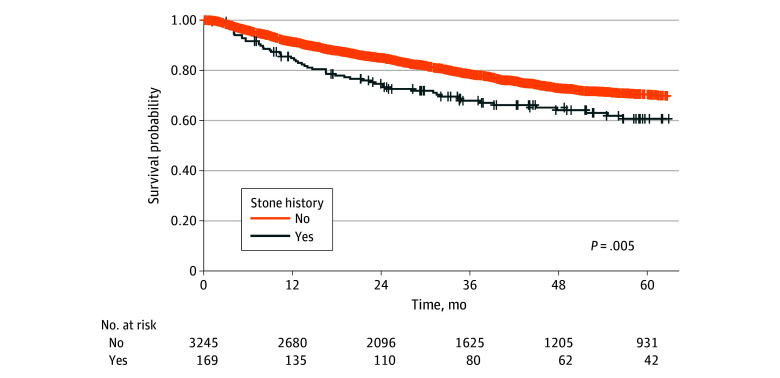
Disease-Free Survival in the Unweighted Cohort, Stratified by Urinary-Stone History Crosses indicate censored patients. Patients with a stone history experienced significantly lower disease-free survival (*P* = .005).

Figure 2 (unweighted) and eFigure 4 in Supplement 1 (overlap-weighted) display DFS for patients with or without a history of urinary stones. In the unweighted analysis, those with stone disease had significantly lower DFS. This disparity persisted after overlap weighting, highlighting the adverse association of stone disease with DFS in UTUC.

### Competing-Risk Analyses

In competing-risk analyses, patients with a history of stones had a clearly higher cumulative incidence of UTUC-specific death, while noncancer deaths were similar between groups (eFigure 5 in Supplement 1). The CSS curves separated early and remained apart through 5 years, whereas the curves for noncancer death ran low and approximately parallel. Consistent with the curves, the Fine-Gray model found that stone history was associated with 78% increased subdistribution hazard of UTUC death (HR, 1.78; 95% CI, 1.30-2.45), independent of other covariates (eTable 2 in Supplement 1). In contrast, bladder recurrence did not differ by stone status: the BRFS cumulative incidence curves overlapped and the Fine-Gray estimate was null (HR, 0.97; 95% CI, 0.73-1.29; *P* = .86) (eFigure 6 in Supplement 1).

## Discussion

In this multi-institutional cohort study of 3414 patients undergoing RNU for UTUC, a history of urinary tract stones was significantly associated with worse oncological outcomes. Specifically, patients with stone history experienced higher rates of metastasis, increased UTUC-specific mortality, and reduced DFS. In directed acyclic graph–based multivariable Cox models, a history of urinary tract stones was independently associated with inferior CSS and shorter DFS. These associations persisted in propensity score overlap–weighted analyses, suggesting robustness to confounding. No statistically significant associations were observed between stone history and OS or BRFS.

Kaplan-Meier survival curves showed that the unfavorable association of stone disease with time-to-event end points, such as DFS and CSS, remained statistically significant even after overlap weighting. This highlights the need for further research to clarify which clinical and pathological factors mediate the adverse outcomes associated with stone disease.

Competing-risk analysis confirmed that the survival disadvantage associated with a history of urinary tract stones was driven by elevated UTUC-specific mortality rather than differences in noncancer deaths. Cumulative incidence curves for noncancer mortality were nearly identical between the stone and no-stone groups, while cancer-specific death curves showed clear separation, indicating higher cancer-related mortality in the stone group. The Fine-Gray subdistribution hazard model supported this interpretation, estimating a 78% increased hazard of UTUC-specific death for patients with stone history, with no significant association between stone history and noncancer mortality. Similarly, bladder recurrence rates were comparable between groups. Cumulative incidence curves for bladder recurrence overlapped throughout follow-up, and the Fine-Gray model yielded a null association, suggesting that stone history does not influence the risk of subsequent bladder tumors.

### Interpretation

Our findings underscore the need for more vigilant surveillance in patients with UTUC and a history of urolithiasis. Stone-related UTUC is characterized by inferior DFS and CSS, pointing to a distinct and potentially more aggressive disease course. Most UTUC-specific deaths occur within 2 to 3 years of diagnosis, highlighting an early high-risk period. As such, more frequent imaging and endoscopic evaluations, as well as earlier or more intensive interventions, should be considered, especially for patients with high-risk features, such as lymphovascular invasion, multifocal disease, or positive margins.

### Shared Metabolic and Genetic Pathways

Smoking has been closely linked to both urinary stone formation and urothelial carcinoma.^17^ Potential mechanisms include vasopressin elevation, oxidative stress, and renal tubular dysfunction, all of which may contribute to stone formation and tumor development.^18^ Several biological processes could account for the intensified aggressiveness of UTUC in patients with urolithiasis. Persistent irritation and inflammation caused by stones can increase urothelial turnover and promote malignant transformation,^19^ while obstruction may delay diagnosis and allow tumors to reach more advanced stages.

Alterations in the calcium channel transient receptor potential cation channel subfamily V member 5 (TRPV5) and the glycoprotein mucin-1 (MUC1) have been associated with both hypercalciuria, which predisposes individuals to kidney stones, and tumor progression. Disrupting TRPV5 may lead to excessive calcium excretion that contributes to stone disease,^20^ and overexpression of MUC1 in UTUC has been linked to higher tumor grade and invasiveness.^21,22^ These observations point to a possible shared molecular network underpinning both stone formation and urothelial carcinogenesis.

### Clinical Implications

The phase 3 POUT trial established that delivering 4 cycles of gemcitabine-platinum chemotherapy within 90 days of radical nephroureterectomy leads to significant improvements in DFS and metastasis-free survival among patients with locally advanced UTUC (pT2-T4 or N+).^23^ Although stone history was not evaluated in POUT, our data indicate that patients with UTUC and prior urolithiasis had notably worse CSS and DFS, suggesting a more aggressive phenotype. While additional randomized studies are needed to confirm these observations, adjuvant chemotherapy could be especially beneficial for patients with UTUC and prior stone disease, given their heightened risks of recurrence and mortality.

### Suboptimal Outcomes With Adjuvant Chemotherapy

In the univariate Cox analysis, adjuvant chemotherapy was significantly associated with worse CSS and DFS, but not OS or BRFS. The observed outcomes could reflect potential selection bias, as patients chosen for adjuvant chemotherapy may present with more advanced or aggressive disease characteristics. Furthermore, these findings highlight the possibility that standard adjuvant chemotherapy regimens might be inadequate for this specific subgroup of patients. Emerging therapeutic options, such as immunotherapy or antibody-drug conjugates, which have demonstrated efficacy in aggressive urothelial cancers,^24^ may therefore be warranted.

### Earlier Management of Urinary Stones and UTUC Outcomes

It remains unclear whether prompt stone removal or more focused metabolic management can lower UTUC risk or enhance survival. Chronic inflammation and obstruction from stones have been proposed as contributors to tumor progression, but evidence is lacking to confirm whether proactive stone management can modify this trajectory. Prospective trials exploring early surgical stone removal, metabolic evaluations, and lifestyle adjustments are needed to determine whether these measures can reduce UTUC occurrence or improve outcomes in susceptible patients.

### Generalizability

Although the overall sample size was large, only a small proportion (4.9%) of patients had a documented history of urinary tract stones, restricting the power to detect associations in this subgroup and limiting the external validity of findings. Overlap weighting improved covariate balance and reduced confounding; however, it confines inference to the region of covariate overlap. As a result, the findings may not be fully generalizable to the broader UTUC population.

### Limitations

This study has some limitations. As a multi-institutional retrospective registry study, it is inherently subject to selection bias and residual confounding, despite the use of prespecified covariate adjustment and propensity score overlap weighting. Differences in diagnostic and treatment pathways for stones, as well as variability in the time between stone development and UTUC detection, could affect staging at presentation.

The exposure of interest, urinary stones history, was abstracted from medical records and coded as a binary variable. Detailed information on timing, laterality, number, size, composition, radiographic features, symptoms, and prior interventions was unavailable, making it impossible to distinguish chronic, obstructive stones from incidental or clinically insignificant calculi. Because registry data were collected after UTUC diagnosis, earlier stone events that were asymptomatic, occurred many years before diagnosis, or were managed at other institutions may have been incompletely recorded or entirely missed. Consequently, some patients with stone history may have been misclassified as having no history, introducing recording or recall bias that would likely bias estimates toward the null and suggest that the true associations may be stronger than observed. In addition, patients with missing covariate data or incomplete follow-up were excluded from the analysis. This exclusion may have led to selection bias, potentially favoring healthier individuals or those with more complete medical documentation. Although the direction of this bias is uncertain, it could attenuate the reported associations. Furthermore, the absence of longitudinal and image-linked data in the registry prevented analysis of the temporal association between stone disease and cancer development. Therefore, causal interpretations should be made with caution.

## Conclusions

In this cohort study of patients with UTUC who underwent RNU, a history of urinary tract stones was associated with higher UTUC-specific mortality and a greater risk of disease recurrence. Although the association with overall survival was less certain after adjusting, these findings suggest that patients with UTUC and urinary tract stone history represented a distinctly high-risk subgroup. Enhanced surveillance and risk-directed therapies may help offset the negative prognostic outcomes associated with stone disease, and future investigations should focus on unraveling the molecular basis of this association and optimizing personalized management for these patients.

## Notes

Supplement 1. **eFigure 1.** Study Flow Diagram **eFigure 2.** Directed Acyclic Graph **eFigure 3.** Cancer-Specific Survival in the Overlap-Weighted Cohort, Stratified by Urinary-Stone History **eFigure 4.** Disease-Free Survival in the Overlap-Weighted Cohort, Stratified by Urinary-Stone History **eFigure 5.** Cumulative Incidence Curve for Cancer-Specific Survival **eFigure 6.** Cumulative Incidence Curve for Bladder Recurrence-Free Survival
eMethods.

Supplement 2.
Data Sharing Statement
