# Supplementary material for: Kidney Stone History and Survival Outcomes in Upper Tract Urothelial Carcinoma
Source: JAMA Netw Open. 2025 Nov 3;8(11):e2541054. doi: 10.1001/jamanetworkopen.2025.41054 (PMC12584037; doi:10.1001/jamanetworkopen.2025.41054)
Supplement: Supplement 1. — eFigure 1. Study Flow Diagram eFigure 2. Directed Acyclic Graph eFigure 3. Cancer-Specific Survival in the Overlap-Weighted Cohort, Stratified by Urinary-Stone History eFigure 4. Disease-Free Survival in the Overlap-Weighted Cohort, Stratified by Urinary-Stone History eFigure 5. Cumulative Incidence Curve for Cancer-Specific Survival eFigure 6. Cumulative Incidence Curve for Bladder Recurrence-Free Survival eMethods. [file jamanetwopen-e2541054-s001.pdf]

Supplemental Online Content

Jong BE, Wu HC, Chen WC, et al. Kidney stone history and survival outcomes in upper tract urothelial carcinoma. *JAMA Netw Open*. 2025;8(11):e2541054. doi:10.1001/jamanetworkopen.2025.41054

eFigure 1. Study Flow Diagram
eFigure 2. Directed Acyclic Graph
eFigure 3. Cancer-Specific Survival in the Overlap-Weighted Cohort, Stratified by Urinary-Stone History
eFigure 4. Disease-Free Survival in the Overlap-Weighted Cohort, Stratified by Urinary-Stone History
eFigure 5. Cumulative Incidence Curve for Cancer-Specific Survival
eFigure 6. Cumulative Incidence Curve for Bladder Recurrence-Free Survival
eMethods.

This supplemental material has been provided by the authors to give readers additional information about their work.

© 2025 Jong BE et al. *JAMA Network Open*.

eTable 1. Complete Patient Characteristic Baseline (N = 3414)

Variables	No stone	Stone	p	SMD
**N**	3245	169		
**Gender (%)**			0.001*	0.275
1 Male	1363 (42.0)	94 (55.6)		
2 Female	1882 (58.0)	75 (44.4)	0.641	0.039
**Age (mean (SD))**	68.21 (10.52)	67.83 (9.34)		
**Risk Factors**				
Smoking (%)			0.003*	0.222
0 No	2711 (83.5)	126 (74.6)		
1 Yes	534 (16.5)	43 (25.4)		
Herbal supplements (%)			0.387	0.088
0 No	3048 (93.9)	162 (95.9)		
1 Yes	197 (6.1)	7 (4.1)		
Family hx (%)			0.195	0.177
0 No	3195 (98.5)	169 (100.0)		
1 Yes	50 (1.5)	0 (0.0)		
ESRD/ CRI (%)			0.233	0.1
0 No	1168 (36.0)	69 (40.8)		
1 Yes	2077 (64.0)	100 (59.2)		
Previous Nephroureterectomy for UC (%)			0.078*	0.2
0 No	3136 (96.6)	168 (99.4)		

© 2025 Jong BE et al. *JAMA Network Open*.

1 Yes	109 (3.4)	1 (0.6)		
Previous renal sparing surgery for UTUC (%)			0.736	0.1
0 No	3229 (99.5)	169 (100.0)		
1 Yes	16 (0.5)	0 (0.0)		
Bladder Cancer (%)			0.048*	0.193
0 No	2984 (92.0)	163 (96.4)		
1 Yes	261 (8.0)	6 (3.6)		
**NxUx Method (%)**			0.158	0.184
1 Open	909 (28.0)	48 (28.4)		
2 Laparoscopic hand-assisted	898 (27.7)	58 (34.3)		
3 Robot assisted	353 (10.9)	12 (7.1)		
4 Laparoscopy	1085 (33.4)	51 (30.2)		
**NxUx Access (%)**			0.823	0.024
6 Transperitoneal	1421 (43.8)	72 (42.6)		
7 Retroperitoneal	1824 (56.2)	97 (57.4)		
**Post-operation Intravesical CT Instillation (%)**			0.079	0.175
0 No	3028 (93.3)	164 (97.0)		
1 Intravesical therapy	217 (6.7)	5 (3.0)		
**Cell Type (%)**			0.429	0.141
1 Urothelial	2917 (89.9)	153 (90.5)		
5 UC with variants	296 (9.1)	16 (9.5)		
Squamous cell carcinoma	142 (48.0)	11 (68.8)		

© 2025 Jong BE et al. *JAMA Network Open*.

Sarcomatoid differentiation	45 (15.2)	2 (12.5)		
Adenocarcinoma	27 (9.1)	1 (6.2)		
Neuroendocrine tumors	7 (2.4)	0 (0.0)		
Mixed-cell type	21 (7.1)	0 (0.0)		
Missing	54 (18.2)	2 (12.5)		
6 Others	32 (1.0)	0 (0.0)		
**Side (%)**			0.117	0.196
1 Left	1654 (51.0)	97 (57.4)		
2 Right	1551 (47.8)	72 (42.6)		
3 Both	40 (1.2)	0 (0.0)		
**Location (%)**			0.021*	0.244
1 Renal pelvis	1374 (42.3)	64 (37.9)		
2 Ureter	1029 (31.7)	44 (26.0)		
3 Bladder cuff	11 (0.3)	0 (0.0)		
4 Multiple locations	831 (25.6)	61 (36.1)	0.002*	0.363
**Size (%)**	831 (25.6)	44 (26.0)		
1 <2cm				
2 ≧2cm	2165 (66.7)	124 (73.4)		
3 Missing	249 (7.7)	1 (0.6)		
**Pathological Stage (%)**			0.913	0.034
0 Stage 0a/0is/I	1347 (41.5)	72 (42.6)		
1 Stage II	617 (19.0)	30 (17.8)		

© 2025 Jong BE et al. *JAMA Network Open*.

2 Stage III/IV	1281 (39.5)	67 (39.6)		
**Grade (%)**			0.078	0.209
1 Low grade	375 (11.6)	24 (14.2)		
2 High grade	2693 (83.0)	142 (84.0)		
3 Missing	177 (5.5)	3 (1.8)		
**CIS (%)**			0.281	0.095
0 No	2505 (77.2)	137 (81.1)		
1 Yes	740 (22.8)	32 (18.9)		
**LVI (%)**			0.24	0.097
0 No	2625 (80.9)	130 (76.9)		
1 Yes	620 (19.1)	39 (23.1)		
**Surgical Margin (%)**			0.884	0.026
0 Free	3109 (95.8)	161 (95.3)		
1 Positive	136 (4.2)	8 (4.7)		
**Pre-operation Urine Cytology (%)**			0.087	0.214
0 Negative	759 (23.4)	26 (15.4)		
1 Atypia	544 (16.8)	34 (20.1)		
2 Positive	809 (24.9)	42 (24.9)		
3 No cytology	1133 (34.9)	67 (39.6)		
**Pre-operative Hydronephrosis (%)**			<0.001*	0.577
0 No	1428 (44.0)	31 (18.3)		
1 Yes	1817 (56.0)	138 (81.7)		

© 2025 Jong BE et al. *JAMA Network Open*.

**Tumor Necrosis (%)**			0.004*	0.214
0 No	2767 (85.3)	130 (76.9)		
1 Yes	478 (14.7)	39 (23.1)		
**Chemotherapy Type for UTUC (%)**			0.525	0.115
0 No	2307 (71.1)	116 (68.6)		
1 Neo-adjuvant	86 (2.7)	3 (1.8)		
2 Adjuvant	598 (18.4)	32 (18.9)		
3 Salvage/Palliative	254 (7.8)	18 (10.7)		
**Follow up OS/CSS (median (Q1, Q3))**	53.71 (23.75, 92.77)	54.8 (22.93, 87.35)	0.113	0.135
**Follow up BRFS/DFS (median (Q1, Q3))**	36.01 (16.69, 65.67)	34.49 (16.46, 59.49)	0.489	0.055
**Residual Bladder Cuff (%)**			0.241	0.097
0 No	2696 (83.1)	134 (79.3)		
1 Yes	549 (16.9)	35 (20.7)		
**Metastasis (%)**			<0.001*	0.245
0 No	3012 (92.8)	144 (85.2)		
1 Yes	233 (7.2)	25 (14.8)		
**Mortality (%)**			0.975	0.009
0 No	1819 (56.1)	94 (55.6)		
1 Yes	1426 (43.9)	75 (44.4)		
**UTUC Mortality (%)**			0.003*	0.223
0 No	2646 (81.5)	122 (72.2)		
1 Yes	599 (18.5)	47 (27.8)		

© 2025 Jong BE et al. *JAMA Network Open*.

**Disease Free (%)**			0.012*	0.199
0 No	838 (25.8)	59 (34.9)		
1 Yes	2407 (74.2)	110 (65.1)		
**Bladder Recurrence (%)**			1	0.005
0 No	2350 (72.4)	122 (72.2)		
1 Yes	895 (27.6)	47 (27.8)		

p-value: **<0.05***

© 2025 Jong BE et al. *JAMA Network Open*.

eTable 2. Fine-Gray Hazard Model

	CSS		BRFS	
	**HR (95% CI)**	**P**	**HR (95% CI)**	**P**
**Stone**				
0 No	1		1	
1 Yes	1.78 (1.3, 2.45)	**<0.001***	0.97 (0.73, 1.29)	0.86
**Gender**				
1 Male			1	
2 Female			0.68 (0.59, 0.77)	**<0.001***
**Age**	1.01 (1, 1.02)	**0.017***	1 (0.99, 1.01)	0.75
**Cell Type**				
1 Urothelial	1			
5 UC with variants	1.42 (1.11, 1.83)	**0.006***		
6 Others	2.03 (1.12, 3.68)	**0.020***		
**Side**				
1 Left			1	
2 Right			0.89 (0.78, 1.01)	0.061
3 Both			0.74 (0.41, 1.33)	0.31
**Location**				
1 Renal pelvis	1		1	
2 Ureter	1.16 (0.94, 1.42)	0.16	1.2 (1.03, 1.4)	**0.021***
3 Bladder cuff	2.74 (1.11, 6.78)	**0.029***	1.42 (0.56, 3.57)	0.46

© 2025 Jong BE et al. *JAMA Network Open*.

4 Multiple	1.46 (1.2, 1.77)	**<0.001***	1.47 (1.26, 1.71)	**<0.001***
**Size**				
1 <2cm	1		1	
2 >=2cm	2.61 (2.02, 3.39)	**<0.001***	1.07 (0.92, 1.24)	0.41
3 Not available	4.84 (3.34, 7.03)	**<0.001***	1.03 (0.75, 1.41)	0.87
**Pathological Stage**				
0 Stage 0a/0is/I			1	
1 Stage II			1.06 (0.9, 1.25)	0.47
2 Stage III/IV			0.74 (0.63, 0.88)	**<0.001***
**Grade**				
1 Low grade	1			
2 High grade	2.84 (1.87, 4.33)	**<0.001***		
3 Not available	1.91 (1.07, 3.41)	**0.029***		
**Risk Factors**				
**Smoking**				
0 No				
1 Yes				
**Previous renal sparing surgery UTUC**				
0 No			1	
1 Yes			2.78 (1.55, 4.99)	**<0.001***
**Bladder Cancer**				
0 No			1	

© 2025 Jong BE et al. *JAMA Network Open*.

1 Yes			1.5 (1.21, 1.84)	**<0.001***
**CIS**				
0 No				
1 Yes				
**LVI**				
0 No	1		1	
1 Yes	2.38 (2, 2.84)	**<0.001***	0.97 (0.81, 1.18)	0.79
**Surgical Margin**				
0 Free	1			
1 Positive	2.84 (2.14, 3.78)	**<0.001***		
**NxUx Method**				
1 Open			1	
2 Laparoscopic hand-assisted			1.15 (0.96, 1.37)	0.12
3 Robot assisted			1.52 (1.18, 1.95)	**<0.001***
4 Laparoscopy			1.39 (1.17, 1.66)	**<0.001***
**NxUx Access**				
6 Transperitoneal			1	
7 Retroperitoneal			1.18 (1.04, 1.35)	**0.013***
**Post-operation Intravesical C/T Instillation**				
0 No			1	
1 Intravesical therapy			1.32 (1.02, 1.71)	**0.033***

P：**<0.05**

© 2025 Jong BE et al. *JAMA Network Open*.

eFigure 1. Study Flow Diagram

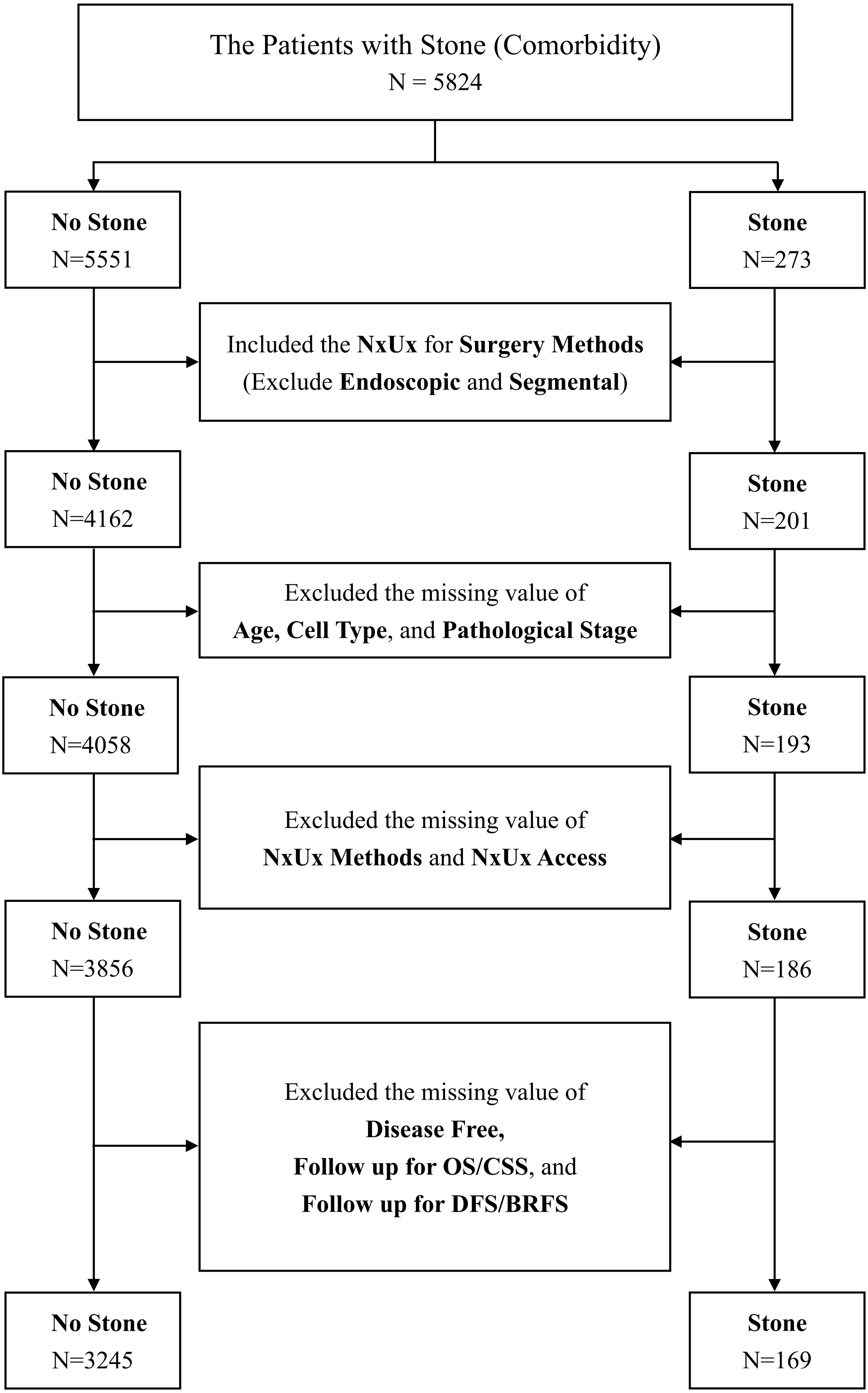

© 2025 Jong BE et al. *JAMA Network Open*.

eFigure 2. Directed Acyclic Graph

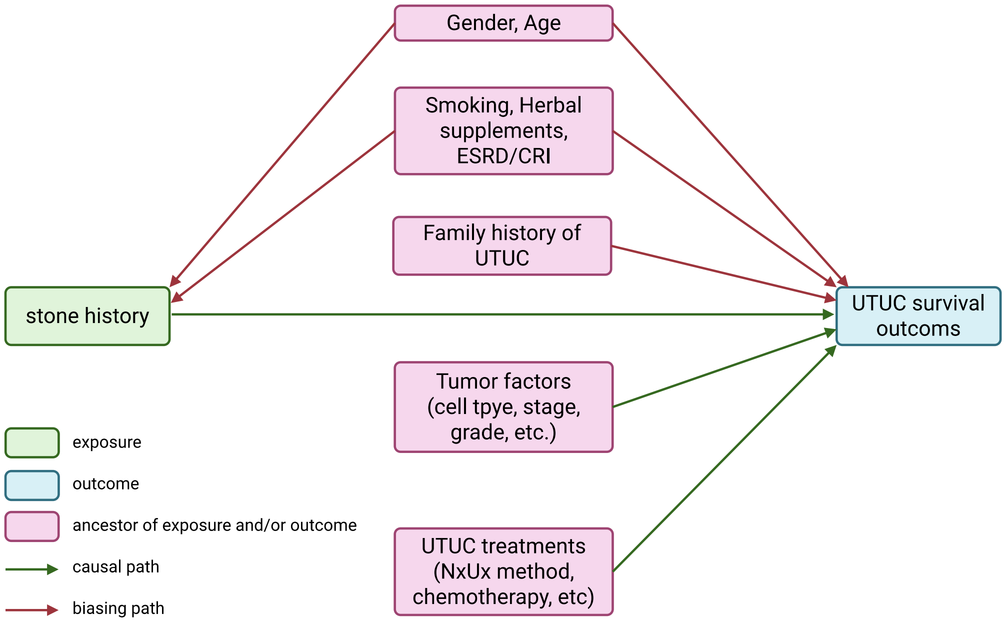

© 2025 Jong BE et al. *JAMA Network Open*.

eFigure 3. Cancer-Specific Survival in the Overlap-Weighted Cohort, Stratified by Urinary-Stone History

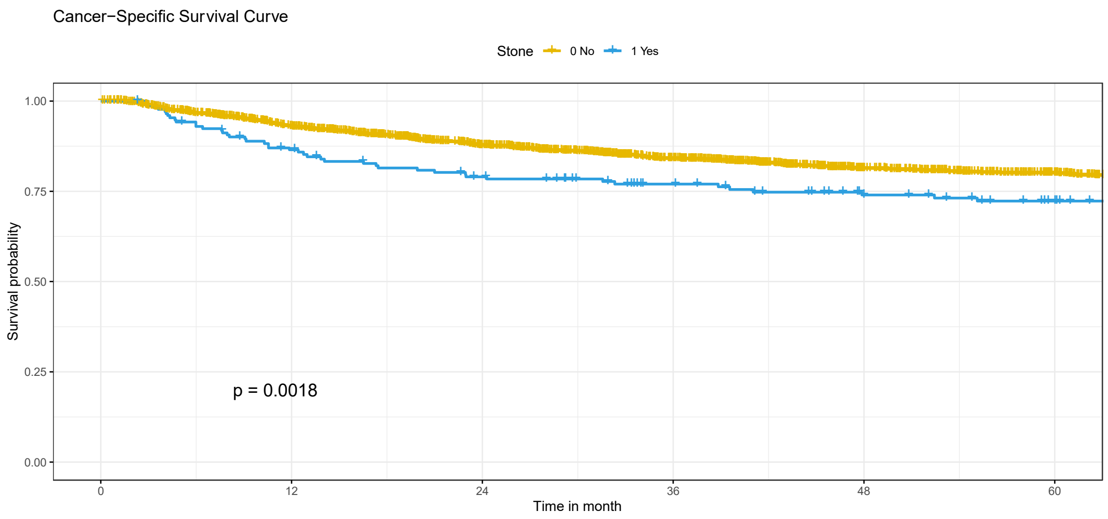

Number at Risk

	0	12	24	36	48	60
0 No	1707	1499	1309	1151	972	854
1 Yes	1707	1430	1260	1079	908	786

eFigure 4. Disease-Free Survival in the Overlap-Weighted Cohort, Stratified by Urinary-Stone History

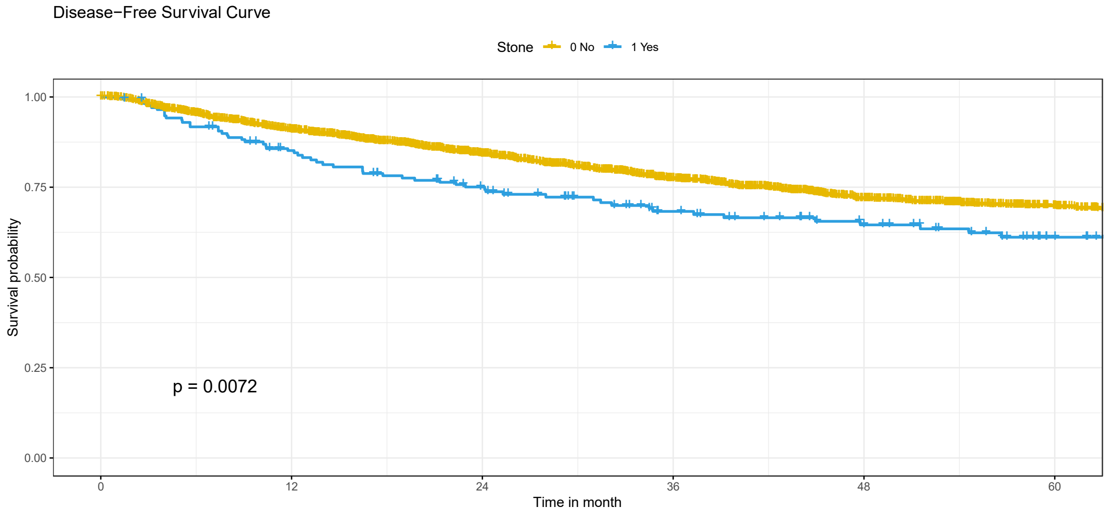

Number at Risk

	0	12	24	36	48	60
0 No	1707	1441	1156	906	692	540
1 Yes	1707	1371	1118	814	631	429

© 2025 Jong BE et al. *JAMA Network Open*.

eFigure 5. Cumulative Incidence Curve for Cancer-Specific Survival

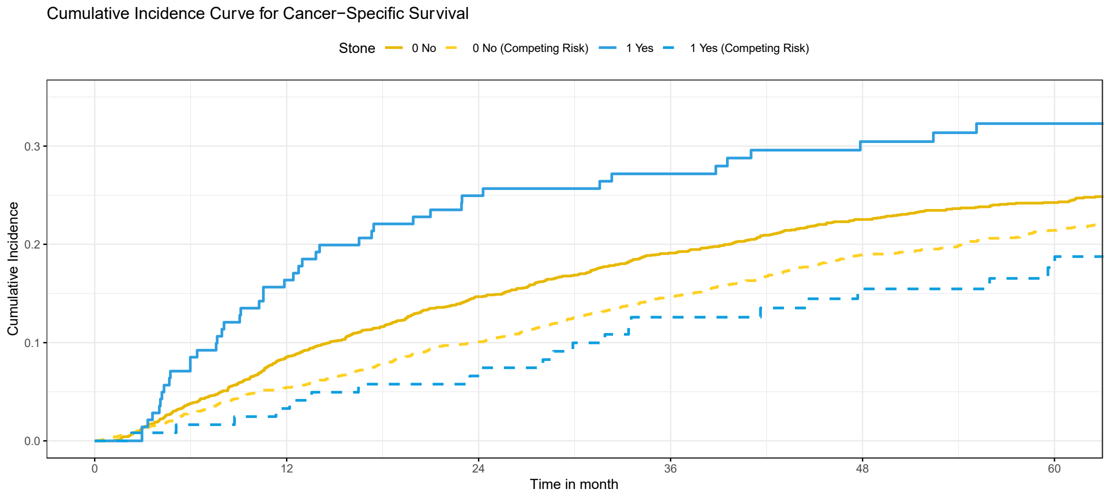

Number at risk

Stone	0	12	24	36	48	60
0 No	2418	2136	1853	1615	1350	1174
0 No (Competing Risk)	2646	2425	2165	1914	1621	1427
1 Yes	141	117	104	93	79	69
1 Yes (Competing Risk)	122	117	112	97	84	74

Time in month

Cumulative number of events

Stone	0	12	24	36	48	60
0 No	0	203	342	435	499	528
0 No (Competing Risk)	0	142	258	364	456	503
1 Yes	0	23	35	38	42	44
1 Yes (Competing Risk)	0	4	8	15	18	20

Time in month

eFigure 6. Cumulative Incidence Curve for Bladder Recurrence-Free Survival

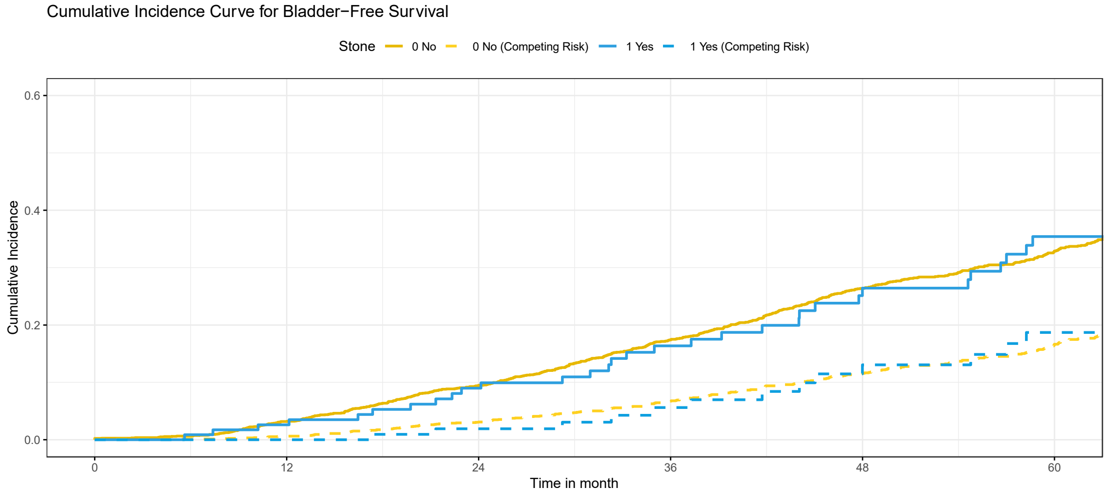

Number at risk

Stone	0	12	24	36	48	60
0 No	2230	2035	1662	1328	995	767
0 No (Competing Risk)	2834	2322	1811	1415	1062	814
1 Yes	116	109	97	73	56	39
1 Yes (Competing Risk)	147	116	96	70	55	38

Time in month

Cumulative number of events

Stone	0	12	24	36	48	60
0 No	5	68	193	329	462	542
0 No (Competing Risk)	1	15	67	128	194	248
1 Yes	0	3	10	17	25	31
1 Yes (Competing Risk)	0	0	2	5	10	13

Time in month

© 2025 Jong BE et al. *JAMA Network Open*.

eFigure 7. Propensity-Score Distributions before Overlap Weighting

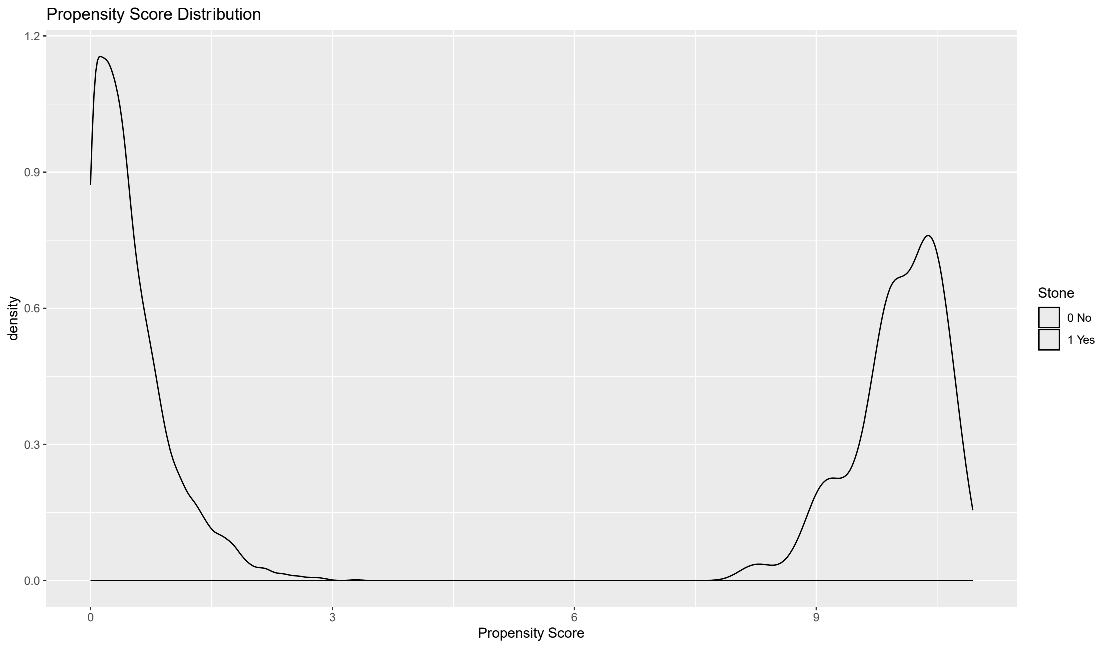

eFigure 8. Propensity-Score Distributions after Overlap Weighting

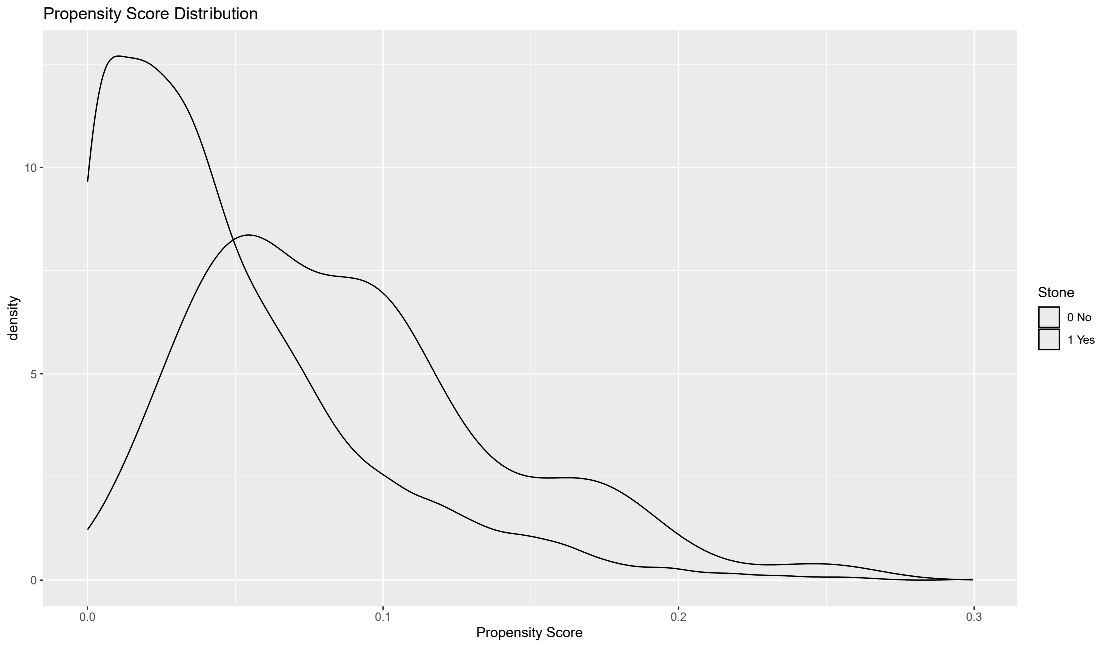

© 2025 Jong BE et al. *JAMA Network Open*.

eFigure 9. Love Plot

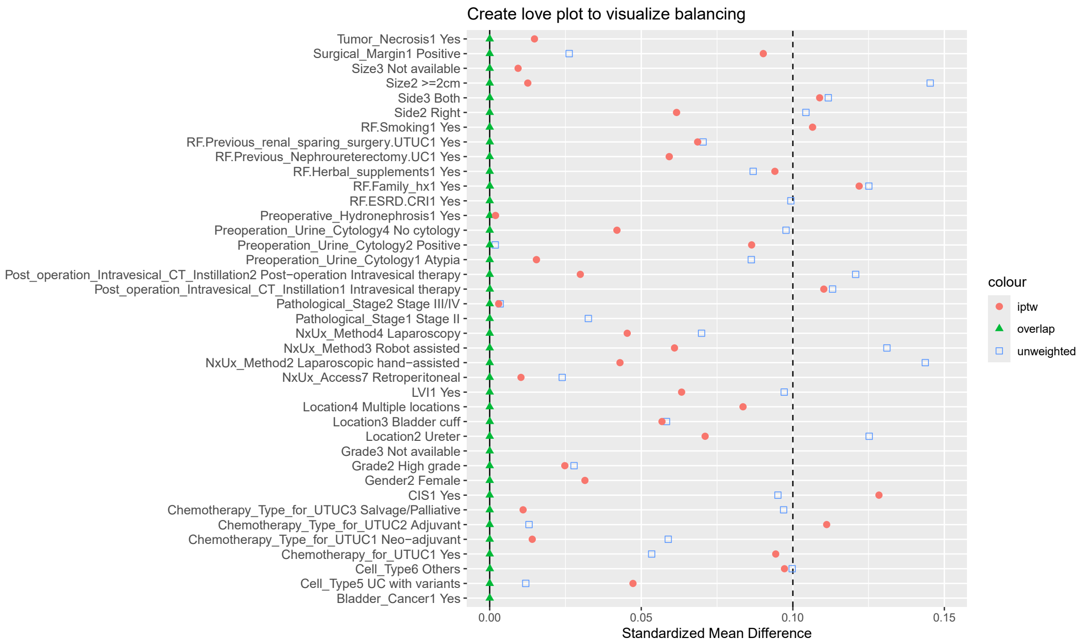

© 2025 Jong BE et al. *JAMA Network Open*.

eMethods

Propensity-Score Overlap Weighting and Cox Proportional Hazards Modeling

Propensity scores for stone history were estimated with multivariable logistic regression including age, sex, calendar year, tumor location, pathological stage and grade, smoking status, Charlson comorbidity index, prior bladder cancer, hospital case volume, and other prespecified clinical variables. Overlap weights were defined as 1 minus the propensity score for patients with a stone history and as the propensity score for those without. Before weighting, propensity-score diagnostics showed substantial baseline imbalance and limited common support, with clearly separated score distributions (eFigure 7). After applying overlap weighting, the distributions converged with substantial overlap (eFigure 8) and covariate balance improved across all variables, with standardized mean differences below 0.10 on the Love plot and most near zero (eFigure 9). Because inverse probability weighting left several covariates above 0.10, overlap weighting was selected for the primary analyses. We then fit overlap-weighted Cox proportional hazards models for OS, CSS, DFS, and BRFS using the *coxph* function from the survival package in R, specifying the overlap weights, setting time zero at radical nephroureterectomy, and censoring at last follow-up. Models included stone history and clinically important covariates, as well as any variables with residual imbalance after weighting, such as pathological stage, tumor

© 2025 Jong BE et al. *JAMA Network Open*.

size, lymphovascular invasion, and margin status. Robust sandwich standard errors were used, and the resulting hazard ratios represent associations in the overlap population.

© 2025 Jong BE et al. *JAMA Network Open*.
