# Supplementary material for: Kidney Stone History and Survival Outcomes in Upper Tract Urothelial Carcinoma
Source: JAMA Netw Open. 2025 Nov 3;8(11):e2541054. doi: 10.1001/jamanetworkopen.2025.41054 (PMC12584037; doi:10.1001/jamanetworkopen.2025.41054)
Supplement: Supplement 2. — Data Sharing Statement [file jamanetwopen-e2541054-s002.pdf]

Data Sharing Statement

Jong. Kidney Stone History and Survival Outcomes in Upper Tract Urothelial Carcinoma.
JAMA Netw Open. Published November 03, 2025. doi:10.1001/jamanetworkopen.2025.41054

Data
Data available: No
